# Mathematical modeling of the microtubule detyrosination/tyrosination cycle for cell-based drug screening design

**DOI:** 10.1371/journal.pcbi.1010236

**Published:** 2022-06-27

**Authors:** Jeremy Grignard, Véronique Lamamy, Eva Vermersch, Philippe Delagrange, Jean-Philippe Stephan, Thierry Dorval, François Fages

**Affiliations:** 1 Pole of Activity Data Sciences and Data Management, Institut de Recherches Servier (IdRS), Croissy-sur-Seine, France; 2 Pole of Activity Cellular Sciences, Institut de Recherches Servier (IdRS), Croissy-sur-Seine, France; 3 Therapeutic Area Neuropsychiatry and Immunoinflammation, Institut de Recherches Servier (IdRS), Croissy-sur-Seine, France; 4 In Vitro Pharmacology Unit, Institut de Recherches Servier (IdRS), Croissy-sur-Seine, France; 5 Team Project Lifeware, Institut National de Recherche en Informatique et Automatique, Inria Saclay, Palaiseau, France; OvGU; Medical Faculty, GERMANY

## Abstract

Microtubules and their post-translational modifications are involved in major cellular processes. In severe diseases such as neurodegenerative disorders, tyrosinated tubulin and tyrosinated microtubules are in lower concentration. We present here a mechanistic mathematical model of the microtubule tyrosination cycle combining computational modeling and high-content image analyses to understand the key kinetic parameters governing the tyrosination status in different cellular models. That mathematical model is parameterized, firstly, for neuronal cells using kinetic values taken from the literature, and, secondly, for proliferative cells, by a change of two parameter values obtained, and shown minimal, by a continuous optimization procedure based on temporal logic constraints to formalize experimental high-content imaging data. In both cases, the mathematical models explain the inability to increase the tyrosination status by activating the Tubulin Tyrosine Ligase enzyme. The tyrosinated tubulin is indeed the product of a chain of two reactions in the cycle: the detyrosinated microtubule depolymerization followed by its tyrosination. The tyrosination status at equilibrium is thus limited by both reaction rates and activating the tyrosination reaction alone is not effective. Our computational model also predicts the effect of inhibiting the Tubulin Carboxy Peptidase enzyme which we have experimentally validated in MEF cellular model. Furthermore, the model predicts that the activation of two particular kinetic parameters, the tyrosination and detyrosinated microtubule depolymerization rate constants, in synergy, should suffice to enable an increase of the tyrosination status in living cells.

## Author summary

Microtubules, cytoskeletal proteins, are involved in essential biological processes such as mitosis, cardiomyocyte contraction and cell motility. The tyrosination reaction, a microtubule post-translational modification is dysregulated in cancer, cardiomyopathies and neuronal diseases. Despite significant advances in recent years, the precise mechanisms regulating the tyrosination cycle and the microtubule dynamics still lack an integrative approach with predictive mathematical modeling. We present a mathematical model of the detyrosination/tyrosination cycle parameterized for neurons and proliferative cells using literature and image-based screening data. The model first explains the inability to increase the tyrosination status by activating the Tubulin Tyrosine Ligase enzyme in those cellular models, second captures the inhibition effect of the Tubulin Carboxy Peptidase enzyme, and third predicts the necessity to combine two drugs to increase the tyrosination status in living cells. Overall, our mathematical model enhances the early drug research by providing critical mechanistic insights and identifying promising targets.

## Introduction

The discovery of new drugs in today’s industrial and scientific environment is a long, costly and risky process [1]. Many failures occur at late stages in the drug discovery pipeline, often in the clinical phase, after years of research and significant investments. The two main reasons why clinical candidates do not reach the market are a lack of efficacy and/or important toxicity [2]. The rational of these failures frequently stems from a limited understanding of the detailed biological processes involved, including:—a comprehensive view of the molecular pathways and targets engaged;—and an exhaustive characterization of the mechanisms of action of drug candidates, including their toxicity. These limitations significantly reduce the investigator ability to rationally select the cellular models best suited for molecule selection and characterization.

Advances in high-content imaging (HCI), data analysis and computational modeling represent tremendous opportunities to enhance the understanding of biological processes, especially early within the drug discovery pipeline for target validation and drug screening design phase [3–6]. Beyond enhancing the understanding of biological processes, these tools can also help challenging the choice of a therapeutic target, deciding on the strategy to directly or indirectly modulate its activity and selecting the most adapted cellular models to maximize the relevance and robustness of the early drug discovery phase.

Microtubules have a wide variety of functions and dynamics depending on the cell type and cell state [7]. This is a consequence of the molecular status of the α/β isotypes of tubulin, the microtubule main subunits, and the remarkable number of post-translational modifications affecting either soluble tubulin or microtubule defining the tubulin code [8,9]. Depending on the tubulin code instance, i.e. specific forms of isotypes and post-translational modifications, the microtubules enable the segregation of chromosomes during mitosis, cell motility and organelles transport [10–13]. Furthermore, the reactions of detyrosination and tyrosination are crucial for neuronal organization, plasticity and differentiation, axon regeneration, protein complexes recruitment, cardiomyocytes contractile functions, and cell proliferation, and are dysregulated in severe diseases such as neurodegenerative disorders, infertility, cardiomyopathies and cancer [14–22]. Consequently, the tubulin code along with its associated signaling pathways represent an important source of potential targets for drug therapies [23–29].

The detyrosination/tyrosination cycle is initiated by the detyrosination of the α/β-tubulin heterodimers, incorporated in microtubules, by Tubulin Carboxy Peptidase (TCP) such as vasohibins (VASH1/VASH2) with the chaperone Small Vasohibin Binding Protein (SVBP) [30–33]. After tubulin depolymerization, the soluble detyrosinated α/β-tubulin heterodimer can be retyrosinated by Tubulin Tyrosine Ligase (TTL) [34,35]. Newly polymerized microtubules are mainly tyrosinated while stable microtubules are detyrosinated [36]. In proliferating cells, microtubules are globally dynamic and tyrosinated, while in neurons, microtubules are generally stable and detyrosinated outside major neuronal structures such as growth cones and dendritic spines which remain highly dynamic and tyrosinated [37,38]. The dynamics and functions of the microtubules are spatially and temporally modulated through regulatory proteins belonging to cross-talking signaling pathways under the control of extracellular and intracellular cues [39–42]. Microtubule-regulating proteins can act directly on microtubules to promote polymerization, depolymerization, stabilization or fragmentation and enable the recruitment and transport of specific protein complexes [43].

Despite significant advances in recent years, the precise mechanisms regulating the tyrosination cycle and ultimately the microtubule dynamics still lack an integrative approach with predictive mathematical modeling. Although previous work was performed on modeling microtubules properties such as trafficking, microtubule dynamics, dynamic instability and microtubule-regulating proteins, there is no available mechanistic mathematical model, to our knowledge, of the tyrosination cycle [44–48]. In this article, we combine computational modeling and high-content imaging data to build a parametrizable mathematical model of the microtubule tyrosination cycle. The part of the computational model concerning microtubule polymerization reactions is based on the linear mode of the microtubule dynamics described in [48]. We present two mathematical models parameterized for neurons and for proliferating cells respectively that enable to understand the modulation effects of key kinetic parameters to increase the tyrosination status in these cellular models.

Tyrosinated tubulin is the product of a chain of two reactions in the cycle: the detyrosinated microtubule depolymerization followed by its tyrosination. The levels of tyrosinated species at equilibrium are thus limited by both reaction rates and activating the tyrosination reaction alone is not effective. Moreover, according to sensitivity analyses and perturbated numerical simulations, decreasing the detyrosination reaction rate constant is predicted to increase the tyrosination status. Using parthenolide, a reference inhibitor of TCP [38], we confirm experimentally the model prediction in MEF cells. Furthermore, in order to design new cell-based screening experiments, especially in neuronal cellular models, the model predicts that increasing, in synergy, two specific biological parameters, the tyrosination and the detyrosinated microtubule depolymerization rate constants, may suffice to increase the tyrosination status in living cells.

## Results

### Generic mathematical model of the microtubule tyrosination cycle

**Fig 1:**
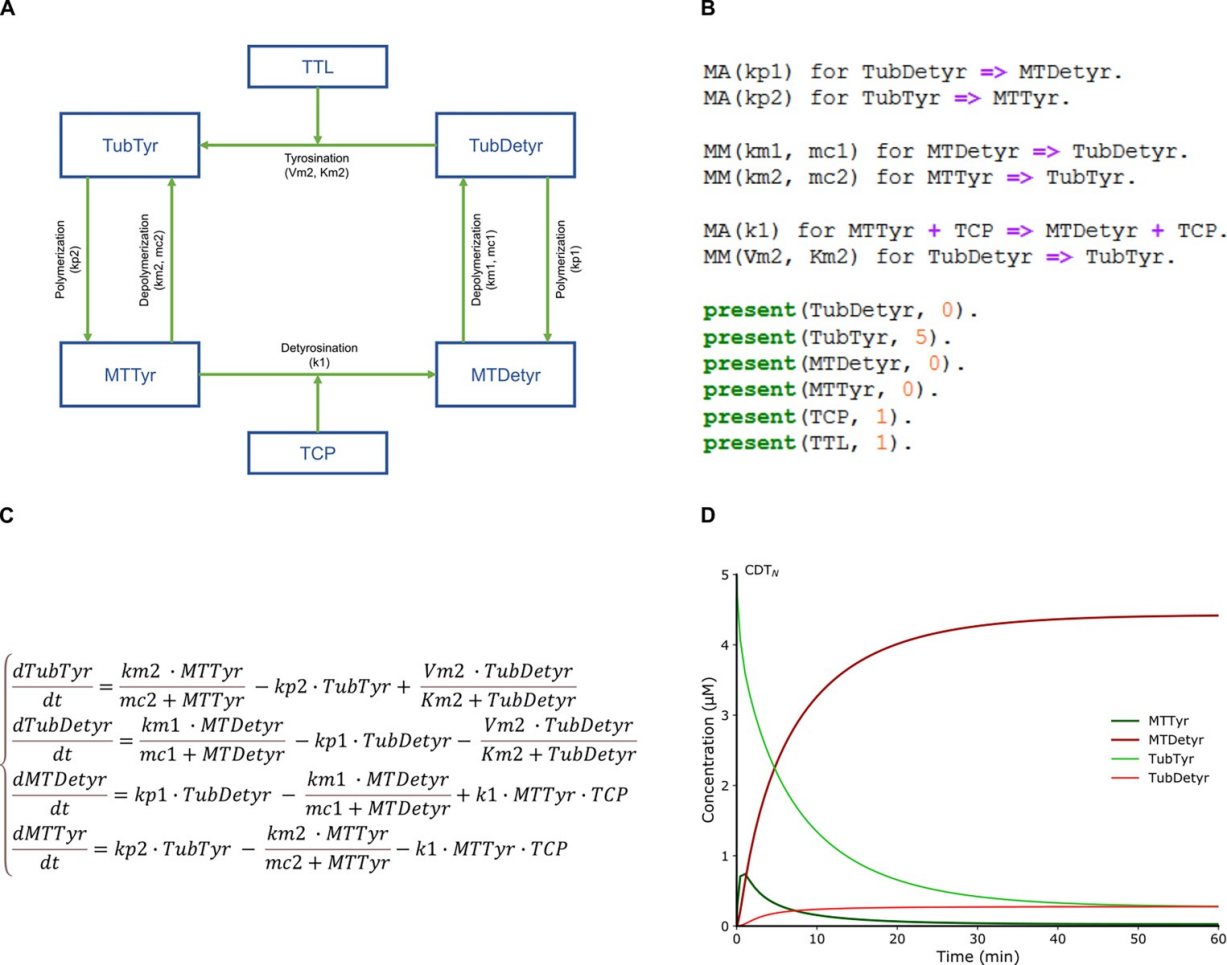
Schematic outline of our mechanistic model building method. (**A**) Influence diagram of the detyrosination/tyrosination cycle. (**B**) Chemical Reaction Network of the detyrosination/tyrosination cycle in BIOCHAM syntax with either mass action law kinetics (MA) or Michaelis-Menten kinetics (MM), plus initial concentrations. (**C**) Parametric Ordinary Differential Equation (ODE) derived from the Chemical Reaction Network. (**D**) Unperturbed numerical simulation of the computational model CDT_N_, parameterized with kinetics values taken from the literature and hypothesis.

The schematic outline of our modeling pipeline (Fig 1) illustrates our model building method. We first design a Chemical Reaction Network (CRN) focusing on the main molecular species and parameters governing the microtubule tyrosination cycle, without taking into account all the other known microtubule-regulating proteins with their associated signaling pathways, due to their high number and unknown kinetics [43,49]. We focus on tubulin and microtubule in their detyrosinated and tyrosinated forms and on the TCP and TTL enzymes as main regulators (Fig 1A). This CRN is formatted in the Systems Biology Markup Language (SBML) [50] and implemented in the Biochemical Abstract Machine (BIOCHAM) software, a modeling environment for systems biology and synthetic biology [51,52]. The BIOCHAM reaction model consists of 6 reactions: 2 polymerization reactions for detyrosinated and tyrosinated tubulin, 2 depolymerization reactions of detyrosinated and tyrosinated microtubule, and the detyrosination and tyrosination reactions catalyzed by the TCP and TTL enzymes respectively (Fig 1B).

The tyrosination reaction is given with a Michaelis-Menten kinetics based on the enzymatic characterization of TTL performed in bovine brain [53] (Fig 1B). When microtubules are detyrosinated, destabilizing proteins, recruited on tyrosinated microtubule, are released from microtubules [54,55]. We chose to associate to the microtubule depolymerization reactions a Michaelis-Menten kinetics to consider this phenomenon without including new species in the mathematical model. The limiting factors for the tyrosinated and detyrosinated microtubule depolymerization reactions are respectively tyrosinated and detyrosinated microtubule (Fig 1B). To our knowledge there is no enzymatic characterization of TCP and the detyrosination reaction is given here with mass action law kinetics (Fig 1B). Such a set of reactions given with rate functions can be interpreted in BIOCHAM by a continuous time Markov chain (stochastic semantics) or by Ordinary Differential Equations (ODE). For the tyrosination cycle model here involving relatively high numbers of molecules we consider the differential semantics (Fig 1C), i.e. the Ordinary Differential Equation (ODE):

$$\left\{ \begin{array}{c} \frac{dTubTyr}{dt}=\frac{km2∙MTTyr}{mc2+MTTyr}-kp2∙TubTyr+\frac{Vm2∙TubDetyr}{Km2+TubDetyr}\\ \frac{dTubDetyr}{dt}=\frac{km1∙MTDetyr}{mc1+MTDetyr}-kp1∙TubDetyr-\frac{Vm2∙TubDetyr}{Km2+TubDetyr}\\ \frac{dMTDetyr}{dt}=kp1∙TubDetyr-\frac{km1∙MTDetyr}{mc1+MTDetyr}+k1∙MTTyr∙TCP\\ \frac{dMTTyr}{dt}=kp2∙TubTyr-\frac{km2∙MTTyr}{mc2+MTTyr}-k1∙MTTyr∙TCP \end{array}\right.$$

The observed difference in the microtubule dynamics and in the tyrosination states between neuronal and proliferating cells are modeled by changing two reaction kinetic parameter values. Our abstract CRN model of the tyrosination cycle (CDT) will thus give rise to two mathematical models: one parameterized for neuronal cellular models (CDT_N_) and one for proliferating cells (CDT_P_).

### Initial concentrations

The initial molecular concentrations of the CDT_N_ and CDT_P_ models are fixed to the same values accordingly to literature data and hypothesis (Table 1). In Fig 2E from [32], authors accessed the detyrosination activity of purified VASH1/SVBP complexes (TCP) on brain microtubules or tubulin dimers using immunoblot. They observed the time evolution of the tyrosination cycle species on four time points (0, 2, 5, 10 minutes), with only tyrosinated species present at the beginning of their experiment. In our computational model, the initial concentration of tyrosinated tubulin is set to 5 μM accordingly to the tubulin concentrations indicated in [32] and in other cells [56,57]. The other initial concentrations of the cycle are set to 0 μM in our simulations since they will not change the equilibrium state of the system (see Multistability analysis in Materials and Methods).

**Fig 2:**
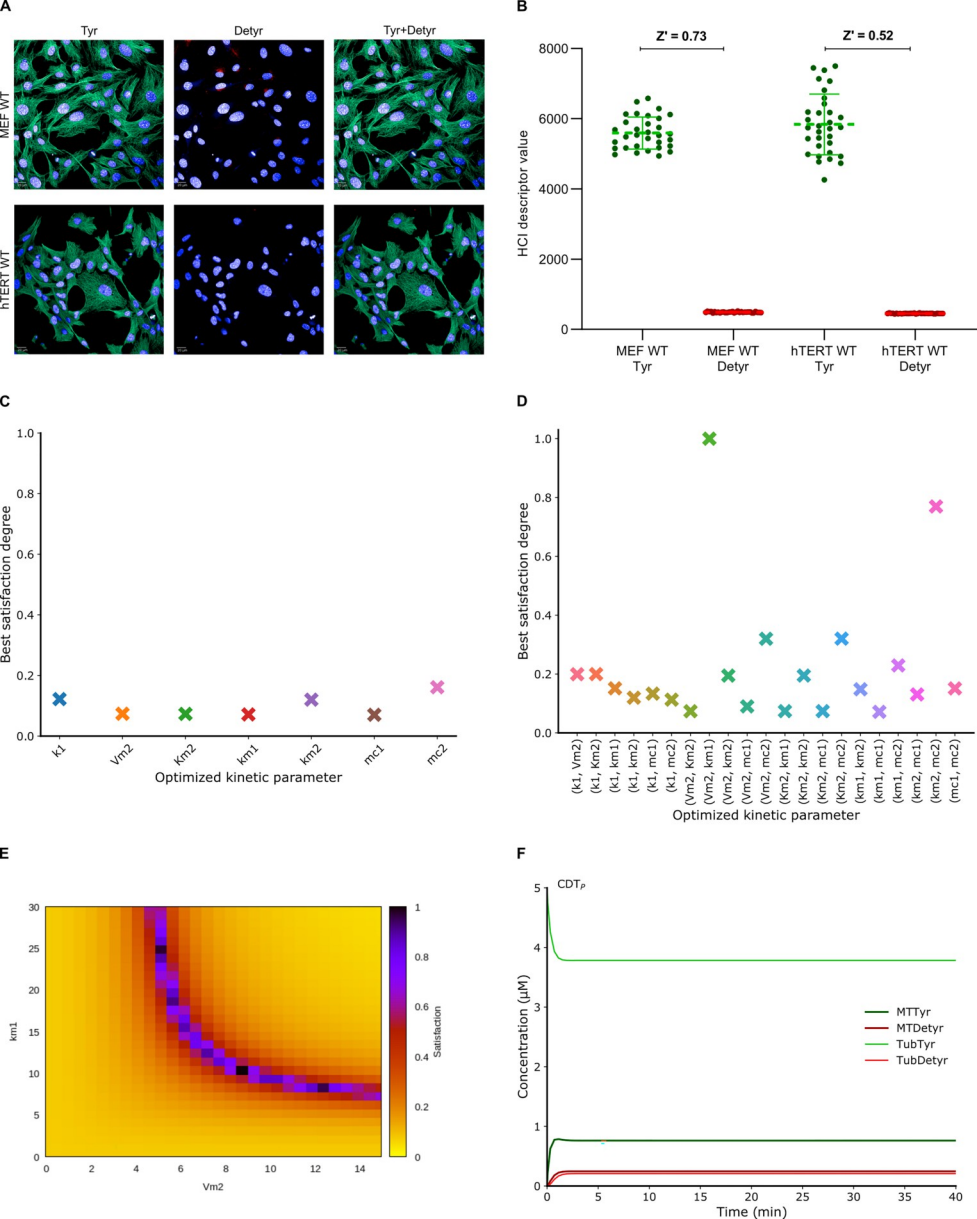
Parameterization of the computational model CDT_P_ with HCI quantification combined with BIOCHAM parameter optimization procedures. (**A**) Representative images of immunostaining of tyrosinated tubulin (Tyr) in green, detyrosinated tubulin (Detyr) in red in MEF cells and hTERT RPE-1 cells (left). Cells were co-stained with Hoechst. Scale bars: 20 μm. (B) Quantification of the tyrosination status by high-content imaging (Right). Tubulin and microtubule are predominantly observed in tyrosinated form (Z’-factor > 0.5). The plotted values are the average of single-cells values ± SD. (C) Best satisfaction degree obtained by the parameter search procedure by varying only one kinetic parameter independently, showing failure to reproduce the observed behaviour. The BIOCHAM command used is: search_parameters(F(Time == 5 /\ Tyr = factor1 * Detyr /\ F(Time == 20 /\ Tyr = factor2 * Detyr)), [0 <= p <= 100], [factor1 -> 10, factor2 -> 10]) where p is the kinetic parameter to optimize. (D) Best satisfaction degree obtained by the parameter search procedure by varying couples of two kinetic parameters simultaneously, showing perfect satisfaction of the specification with one couple of parameters only: (*V*_*m*2_, *k*_*m*1_). The BIOCHAM command used is: search_parameters(F(Time == 5 /\ Tyr = factor1 * Detyr /\ F(Time == 20 /\ Tyr = factor2 * Detyr)), [0 <= p1 <= 100, 0 <= p2<= 100], [factor1 -> 10, factor2 -> 10]) where p1 and p2 are two kinetic parameters to optimize. (E) Landscape of the satisfaction degree obtained by scanning the parameter values of the couple (*V*_*m*2_, *k*_*m*1_). The BIOCHAM command used to obtain the landscape is: scan_parameters(F(Time == 5 /\ Tyr = factor1 * Detyr /\ F(Time == 20 /\ Tyr = factor2 * Detyr)), (0 <= Vm2 <= 15), (0 <= km1 <= 30), [factor1 -> 10, factor2 -> 10], resolution:30). (F) Unperturbed numerical simulation of the CDT_P_ model showing the maintenance of a high level of tyrosination. The FO-LTL formulae used to infer the new parameter values for has been updated to infer new parameters with minimal difference from their original values from the CDT_N_ model: search_parameters(F(Time == 5 /\ Vm2 = VarVm2 /\ km1 = Varkm1 /\ Tyr = factor1 * Detyr /\ F(Time == 20 /\ Tyr = factor2 * Detyr)), [0 <= Vm2 <= 15, 0 <= km1 <= 30], [VarVm2 -> 0.2, Varkm1 -> 0.478, factor1 -> 10, factor2 -> 10]).

**Table 1:** Parameter values of the computational models.

Description	Parameter	Unit	CDT_N_	CDT_P_
*Initial concentration*				
Detyrosinated tubulin		μM	0	0
Tyrosinated tubulin		μM	5	5
Detyrosinated microtubule		μM	0	0
Tyrosinated microtubule		μM	0	0
Tubulin Tyrosine Ligase (TTL)		μM	1	1
Tubulin Carboxy Peptidase (TCP)		μM	1	1
*Reactions rates*				
Polymerization of detyrosinated microtubule	*k*_*p*1_	μM^-1^.min^-1^	0.975	0.975
Polymerization of tyrosinated microtubule	*k*_*p*2_	μM^-1^.min^-1^	0.975	0.975
Depolymerization of detyrosinated microtubule	*k*_*m*1_ *mc*_1_	min^-1^ μM	**0.478** 2.75	**11.74** 2.75
Depolymerization of tyrosinated microtubule	*k*_*m2*_ *mc*_2_	min^-1^ μM	4.78 0.48	4.78 0.48
Detyrosination	*k*_1_	μM^-2^.min^-1^	1	1
Tyrosination	*V*_*m*2_ *K*_*m*2_	min^-1^ μM	**0.2** 1.9	**7.70** 1.9

### CDT_N_ model built with reaction kinetic values from the literature

The polymerization rate constants, *k*_*p*1_, *k*_*p*2_, the tyrosinated microtubule depolymerization rate constant, *k*_*m*2_, and the tyrosination rate constants, *V*_*m*2_, *K*_*m*2_, are taken from the literature [45,53]. We assume here that the depolymerizing rate constant of detyrosinated microtubule, *k*_*m*1_, is ten times smaller than for tyrosinated microtubule *k*_*m*2_. This assumption first comes from references indicating that detyrosinated microtubule are more stable than tyrosinated microtubule [36–38]. Moreover, it is established that the half-life of tyrosinated microtubule is of the order of minutes while the half-life of detyrosinated microtubule is of the order of hours [58–60]. That difference by one order of magnitude in the microtubule half-lives is reflected in our model by the choice of a depolymerizing rate constant for detyrosinated microtubule (*k*_*m*1_) ten times smaller than for tyrosinated microtubule (*k*_*m*2_). The kinetic parameters *mc*_1_ and *mc*_2_ correspond to Michaelis constants for the detyrosinated and tyrosinated microtubule depolymerization reactions, and without direct experimental data, their values are inferred using a parameter search procedure to obtain the known tyrosinated microtubule half-life of the order of 5 minutes [58–60] (see Parameter search procedure in Materials and Methods). We do not have any prior value for the detyrosination reaction rate constant, *k*_1_, but we set its value to 1 μM^-2^.min^-1^ knowing that TCP should act slowly on microtubule [61].

With these parameter values, the numerical integration of the ODE associated to the CDT_N_ model gives the evolution of all the molecular concentrations over time (Fig 1D). These results are in accordance with the detyrosination activity of the VASH1/SVBP complex on the tyrosination cycle species which has already been quantified in related work [32]. The temporal evolution of the molecular species [32], are consistent with the numerical simulation of the CDT_N_ model (Fig 1D). Indeed, the tyrosinated species are almost absent after five minutes [32]; detyrosinated microtubule is the main molecular species at steady state while detyrosinated tubulin increases slightly over time [32]. Furthermore, the half-lives of tyrosinated and detyrosinated microtubules are of the order of minutes and hours respectively (Fig 1D) which is consistent with literature data [32,58–60,62].

### CDT_P_ model obtained by fitting the CDT_N_ model to experimental data

We performed HCI experiments to quantify the tyrosination status in hTERT RPE-1 and MEF cells (Figs 2A, 2B, S1 and S1 Data). Tubulin and microtubule are found to be predominantly tyrosinated (Fig 2B and S1 Data). The observed ratio of fluorescence of tyrosinated over detyrosinated species is greater by a tenfold ratio in those cellular models (Fig 2B and S1 Data).

Our goal is thus to find the minimal changes in the CDT_N_ model that make the system stabilize with a tenfold ratio of tyrosinated over detyrosinated species no later than five minutes knowing that the tyrosinated microtubule half-life is of the order of minutes [58–60]. We formalized that constraint in quantitative temporal logic and used BIOCHAM software to search for parameter values to reproduce that observed behavior by continuous optimization (see Parameter search procedure in Materials and Methods).

The polymerization rate constants (*k*_*p*1_, *k*_*p*2_) are assumed to be the same in the cellular models under study and are not optimized, since the tyrosination status has no effect on the polymerization capability of tubulin and the polymerization rates of tyrosinated and detyrosinated tubulin are known to be similar [63,64].

Interestingly, by trying first to change one kinetic parameter only, in the CDT_N_ model, none of the optimization runs could reproduce the observed behavior (Fig 2C). This is a strong indication, though not a formal proof, that the desired behavior cannot be obtained by changing one parameter only.

Then, optimization runs were performed on all pairs of kinetic parameters. Remarkably, the optimization procedure successfully satisfied the temporal specification for one single pair of kinetic parameters: (*V*_*m*2_, *k*_*m*1_) (Fig 2D). The modulation of the tyrosination reaction (*V*_*m*2_) in synergy with the modulation of the detyrosinated microtubule depolymerization reaction (*k*_*m*1_) thus appears sufficient to reproduce the increase of tyrosination status observed experimentally in proliferative cells. Though not particularly intuitive, and thus especially instructive, this computational result can be compared to some known facts from literature that TTL acts very quickly on tubulins, reflected here by change on *V*_*m*2_ and microtubules are very dynamical in proliferative cells, reflected here by change on *k*_*m*1_ [55].

It is worth remarking that the pair of kinetic parameters (*km*_2_, *mc*_2_), though failing to satisfy the temporal specification, could nevertheless achieve a satisfaction degree of 0.76 (Fig 2D). In these optimized solutions, the system appears always slower to stabilize (twenty minutes), and the inferred values do not seem biologically realistic since *mc*_2_ from (*k*_*m*2_, *mc*_2_) gets a small value in 10^−8^ added to a concentration MTTyr up to 10^−2^ making in effect the reaction independent of the reactant in that case.

The changes limited to *V*_*m*2_ and *k*_*m*1_ can fully satisfy the specification, yet with many solutions. In order to visualize the set of solutions, we scanned parameter values for the couple (*V*_*m*2_, *k*_*m*1_) in reasonable ranges and visualized the satisfaction degree landscape of our behavioral specification (Fig 2E). The landscape indicates that the formula seems fully satisfied along a one-dimensional curve indicating an infinite set of solutions for this pair of parameters (Fig 2E). We performed a new parameter optimization to choose the values minimizing the difference to the original values. This gave the new values of *V*_*m*2_ and *k*_*m*1_ chosen for our CDT_P_ model of proliferative cells, representing an increase by 24.56 fold of *k*_*m*1_ and an increase by 38.54 fold of *V*_*m*2_ (Table 1). This minimal change with respect to the CDT_N_ model suffice to reproduce by simulation the experimental observation that the tyrosinated molecular species stabilize around five minutes at a greater concentration than detyrosinated molecular species by a factor ten (Fig 2F).

### TTL activation cannot significantly increase the tyrosination status in living cells

**Fig 3:**
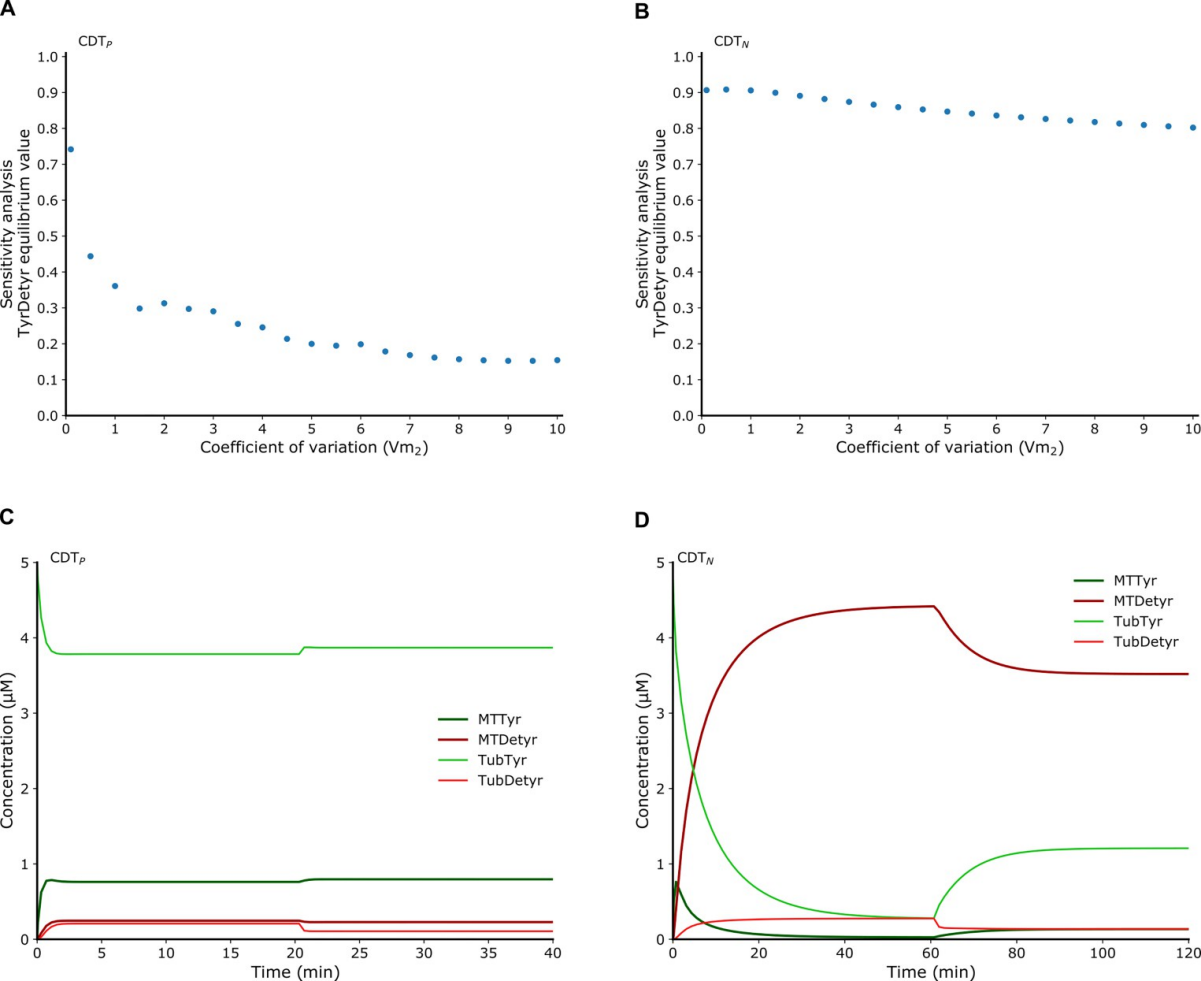
Mathematical model predictions of the tyrosination reaction activation in proliferative and neuronal cells explaining failures of compound screening. (**A**) Sensitivity analysis of the equilibrium value of TyrDetyr obtained for different coefficients of variation of the kinetic parameter *V*_*m*2_ in the computational model CDT_P_, indicating a tolerance of five hundred percent for the parameter *V*_*m*2_ before TyrDetyr deviates from its equilibrium state by eighty percent. The BIOCHAM command used is: sensitivity(F(G(TyrDetyr = x)), [Vm2], [x -> 10], robustness_coeff_var: c), where c is the robustness coefficient value. (**B**) Similar sensitivity analysis in the computational model CDT_N_, indicating a tolerance of five hundred percent for the parameter *V*_*m*2_ before TyrDetyr deviates from its equilibrium state by fifteen percent. The BIOCHAM command used is: sensitivity(F(G(TyrDetyr = x)), [Vm2], [x -> 0.065386], robustness_coeff_var: c) where c is the robustness coefficient value. **(C)** Perturbed numerical simulation in the model CDT_P_. The tyrosination rate constant *V*_*m*2_ is increased at 20 units of time (min) by a factor ten. The numerical simulation shows that the tyrosination status do not increase. (**D**) Perturbed numerical simulation in the model CDT_N._ The tyrosination rate constant *V*_*m*2_ is increased at time 60 (min) by a factor ten. The numerical simulation shows that tyrosinated species slightly increase but are not greater than detyrosinated species at steady state.

We use the CDT_P_ and CDT_N_ computational models to understand the effect of the activation of the TTL enzyme on the tyrosination status in proliferative and neuronal cellular models. First, parameter sensitivity analysis can be used to determine the sensitivity of the tyrosinated species to the tyrosination rate constant *V*_*m*2_ using BIOCHAM (see Robustness measure procedure in Materials and Methods). The sensitivity indices of the CDT_P_ and CDT_N_ models indicate a tolerance of five hundred percent for the parameter *V*_*m*2_, before the tyrosination status deviates from their equilibrium state by eighty and fifteen percent, respectively (Fig 3A and 3B). This indicates that increasing the tyrosination status in this way would necessitate: a very high modulation of the tyrosination rate constant *V*_*m*2_ in neuronal cells which may not be feasible pharmacologically, and a moderate modulation in proliferative cells. Therefore, we further simulate the addition of an activator that could increase the tyrosination reaction by a factor ten in the computational models by increasing the tyrosination rate constant *V*_*m*2_ after the systems have reached their steady state (Fig 3C and 3D). In both models, tyrosinated species increased slightly without exceeding detyrosinated species (Fig 3C and 3D). Increasing the tyrosination reaction rate constant is thus not sufficient in our models to trigger a significant increase of the tyrosination status.

These observations can be explained because tyrosinated tubulin is the product of a chain of two reactions in the cycle: the detyrosinated microtubule depolymerization followed by its tyrosination (Fig 1A). The level of tyrosinated species at equilibrium is thus limited by both reaction rates and activating the tyrosination reaction alone is not effective. In the CDT_P_ model, the level of detyrosinated tubulin remains very low, and in the CDT_N_ model, the level of detyrosinated microtubule remains predominant.

It may be worth mentioning that the obtention of this kind of explanation was our main motivation for developing a mechanistic modeling approach. Indeed, through a cell-free high-throughput screen using a proprietary chemical library, we had identified four compounds increase the tyrosination status of tubulin C-terminals by activating the TTL enzyme (S2 Fig). These active compounds were screened for validation in cell-based high-content screens using proliferating cells, here MEF cells, and hiPSC derived neurons (S3 Fig). However, none of these compounds triggered a significantly increase of the tyrosination status compared to the control conditions (S4 and S5 Figs). The difference in compound activities between the cell-free (S2 Fig) and cell-based (S4 and S5 Figs) assays were the primary motivation of our mechanistic centric approach. The computational models rationalize the lack of activity of proprietary screened compounds in proliferating and neuronal cellular models, by providing one mechanistic reason for the incapacity to directly increase the tyrosination status pharmacologically by activating TTL only, in cells.

### Prediction of the effect of detyrosination inhibition experimentally validated

**Fig 4:**
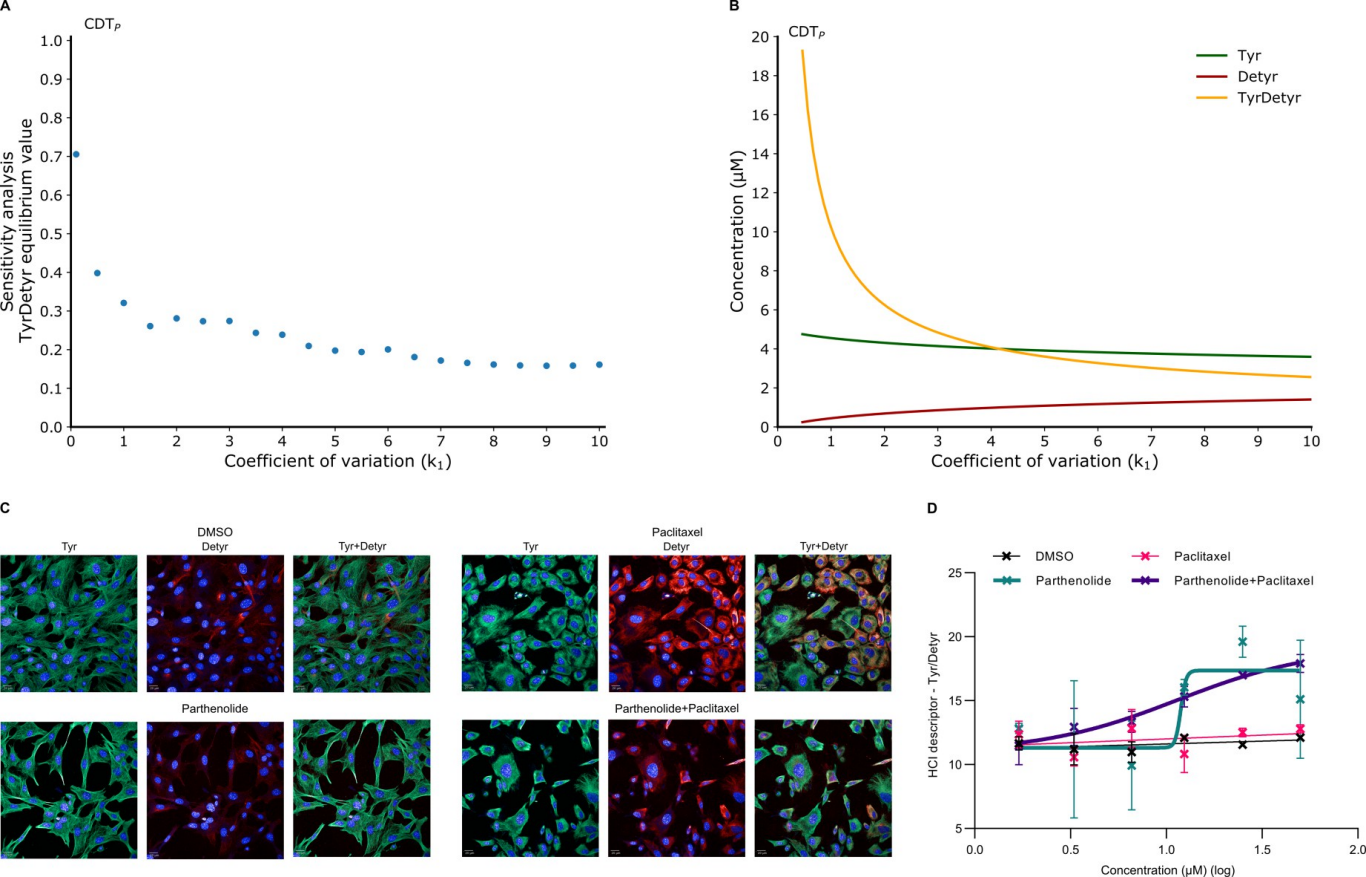
Mathematical model predictions of the detyrosination inhibition with experimental validation in proliferative cells. (**A**) Sensitivity analysis of the equilibrium value of TyrDetyr obtained for different coefficients of variation of the kinetic parameter *k*_1_ in the computational model CDT_P_, indicating that the equilibrium value of TyrDetyr is sensitive for strong variation of *k*_1_. The BIOCHAM command used is: sensitivity(F(G(TyrDetyr = x)), [k1], [x -> 10], robustness_coeff_var: c). where c is the robustness coefficient value. (**B**) Dose response diagram from the CDT_P_ model by varying the kinetic parameter *k*_1_. The BIOCHAM commands used are: change_parameter_to_variable(k1), dose_response(k1, 0, 10, time:100, show:TyrDetyr). The BIOCHAM command draws a dose-response diagram by linear variation of the initial concentration (the dose) of the input object, here *k*_1_, and plotting the output object (the response), here the molecular species: Tyr, Detyr and TyrDetyr, showing an increase of the tyrosination status with a decrease of *k*_1_. (**C)** Compounds screening in dose response of the chemical compound parthenolide. Representative images showing immunostaining of Tyr (green) and Detyr (red) on MEF cells pretreated with or without paclitaxel. Compound concentrations: Parthenolide (1.2 μM (log)), Paclitaxel (5 μM), Parthenolide + Paclitaxel (1.2 μM (log) + 5 μM). Cells were co-stained with Hoechst. Scale bars represent 20 μm. There was no extraction for free tubulin and the visualization of co-localization is potentially impacted. (**D**) Dose response diagrams of the tyrosination status from 4 conditions: DMSO, Paclitaxel, Parthenolide and Parthenolide+Paclitaxel, showing that the tyrosination status increase by inhibiting the detyrosination reaction. Parthenolide concentrations are indicated on the x-axis. Paclitaxel concentration is fixed to 5 μM. We observe that the parthenolide and DMSO error bars overlap at concentrations above 1.5 μM (log). At this parthenolide concentration, the cell morphologies are indeed altered, cytoplasms are reduced, and the cells appear to be highly stressed.

The CDT_P_ model can be further investigated by parameter sensitivity analyses to determine the sensitivity of the tyrosinated species to the detyrosination rate constant *k*_1_. The sensitivity indices of the CDT_P_ model indicates a tolerance of one hundred percent for the parameter *k*_1_ before the tyrosination status deviates from its equilibrium state by seventy percent (Fig 4A). Sensitivity analysis indicates that the tyrosination status can be modulated by a strong modulation of the detyrosination rate constant. Therefore, we simulate the addition of an inhibitor of the detyrosination reaction that could decrease the detyrosination activity, mimicking a dose response experiment in the CDT_P_ model by decreasing the detyrosination rate constant *k*_1_ after the system reaches its steady state (Fig 4B). In the CDT_P_ model, the ratio between tyrosinated and detyrosinated species increases with a decrease of *k*_1_ (Fig 4B). Decreasing the detyrosination reaction is thus predicted to trigger an increase of the tyrosinated species.

We have performed HCI experiments to validate the model prediction using the reference inhibitor of TCP, parthenolide, on MEF cells in dose response [38] (Figs 4C, 4D, S1 and S1 Data). The use of parthenolide increases the tyrosination status (Fig 4D). HCI experiments (Fig 4D) confirm the CDT_P_ model prediction (Fig 4B) of the effect of inhibiting the detyrosination reaction on the tyrosination status.

Interestingly, an increase of *k*_1_ in the model (Fig 4B) induces a decrease of the ratio Tyr/Detyr but does not induce a sigmoidal response as observed experimentally by increasing parthenolide (Fig 4D). We can hypothesize from these observations and our previous modeling assumptions, that parthenolide has a sigmoidal effect on the enzyme TCP while the action of TCP, carried in the computational model by the parameter *k*_1_, has a smooth action on tyrosinated microtubule.

Inhibiting the detyrosination reaction alone is therefore effective to increase the tyrosination status. Our computational models thus predict and capture the effect of inhibiting the enzyme TCP [38]. Those predictions are validated with high-content imaging experiments and literature data and provide a mechanistic explanation of the capacity to directly increase the tyrosination status pharmacologically by inhibiting TCP only.

### Model predictions for new drug combinations and neuronal applications

**Fig 5:**
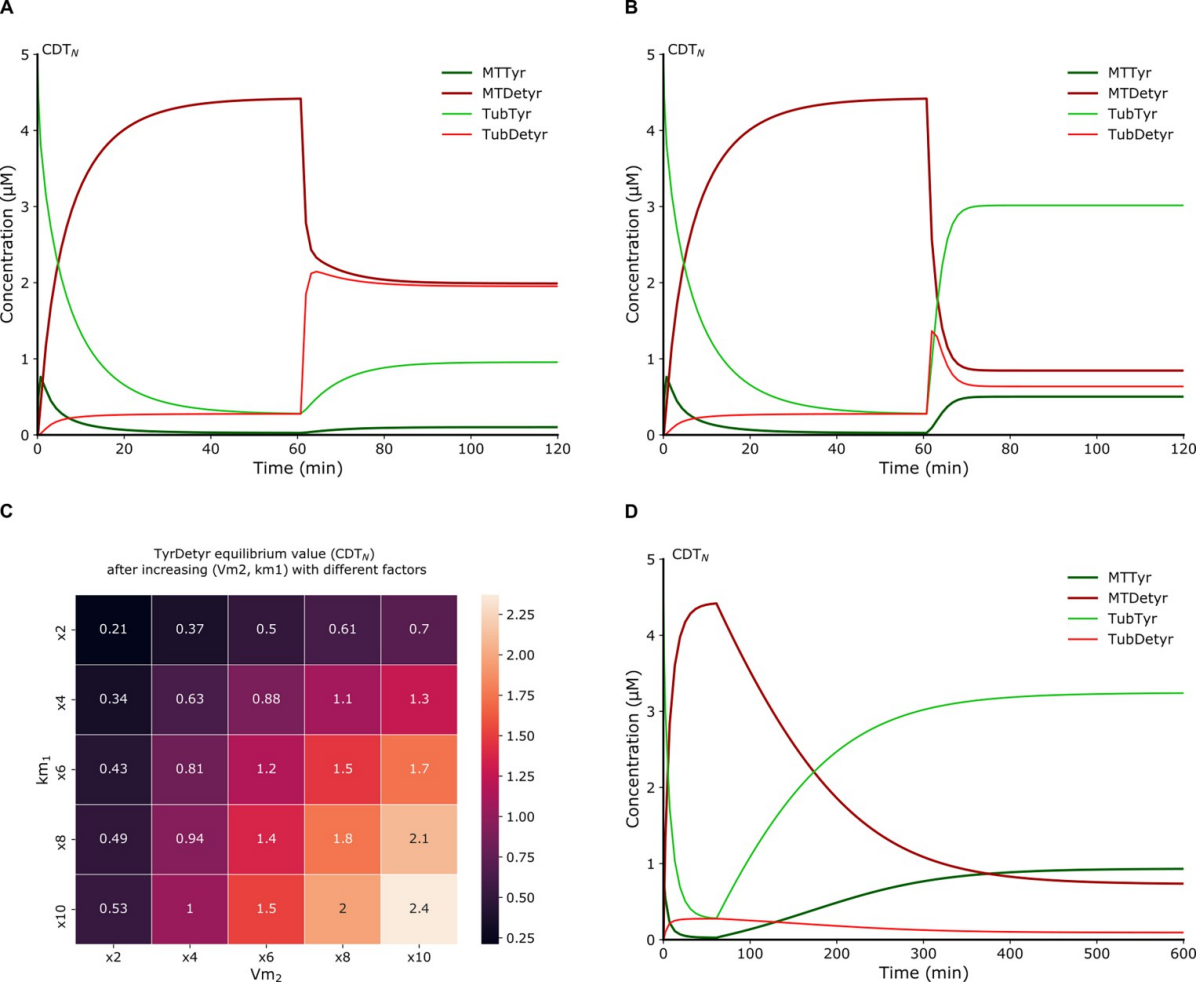
Prediction of drug combinations for potential new screen for neurodegenerative disorders. (**A**) Perturbed numerical simulation in the model CDT_N_. The detyrosinated microtubule depolymerization rate constant *k*_*m*1_ is increased by a factor ten at 60 units of time (min). The numerical simulation shows a slight increase of tyrosinated species suggesting that when the system reached its steady state, increasing the detyrosinated microtubule depolymerization reaction alone does not enable an increase of the tyrosination status. (**B**) Perturbed numerical simulation in the model CDT_N_. The detyrosinated microtubule depolymerization rate constant *k*_*m*1_ and the tyrosination rate constant *V*_*m*2_ are increased at 60 units of time (min) by a factor ten. The numerical simulation shows that the level of tyrosinated species become quickly larger than detyrosinated species. Increasing in synergy the tyrosination and detyrosinated microtubule depolymerization reactions is predicted to be sufficient to trigger a significant increase of the tyrosinated species. (**C**) Prediction of drug combinations combining an increase of the tyrosination rate constant (*V*_*m*2_) and the detyrosinated microtubule depolymerization rate constant (*k*_*m*1_) by different factors showing a synergistic effect to increase the tyrosination status. **(D)** Perturbed numerical simulation in the model CDT_N_. The detyrosination rate constant *k*_1_ is decreased by a factor one hundred at 60 units of time (min). The numerical simulation shows that tyrosinated species slowly increase and become predominant at steady state.

In the perspective of designing new screening experiments in neuronal cellular models, we investigate the CDT_N_ model. As presented, tyrosinated tubulin is the product of a chain of two reactions in the cycle: the detyrosinated microtubule depolymerization followed by its tyrosination. Activating only one reaction is not efficient. The effect of activating the detyrosinated microtubule depolymerization can be investigated by simulating the addition of an activator by increasing the rate constant *k*_*m*1_ after the system has reached its steady state. In these conditions, a slight increase of tyrosinated species is observed (Fig 5A), suggesting that when the system reaches its steady state, increasing the detyrosinated microtubule depolymerization rate constant alone does not enable an increase of the tyrosination status. We also observe an important increase of detyrosinated tubulin which suggests that a pull of tubulins becomes available to reentering the tyrosination cycle (Fig 5A).

Now, one can simulate a prospective drug combination by increasing detyrosinated microtubule depolymerization and tyrosination rate constants (*V*_*m*2_, *k*_*m*1_) in synergy after the system reaches its steady state (Fig 5B). The level of tyrosinated species becomes quickly larger than detyrosinated species (Fig 5B). Increasing in synergy the tyrosination and detyrosinated microtubule depolymerization reactions is thus predicted to trigger a significant increase of the tyrosinated species. Biologically, increasing depolymerizing factors should increase detyrosinated microtubule depolymerization, and allow tubulin to be directly available for retyrosination, while at the same time, increasing tyrosination activity should increase the probability of tubulin to be tyrosinated. It is worth noting that for different parameter increases of the tyrosination activity (*V*_*m*2_) and detyrosinated microtubule depolymerization (*k*_*m*1_), an increase of the tyrosination status can be observed (Fig 5C).

In other work, inhibition of the detyrosination reaction in neurons have been performed experimentally [25]. In particular, the inhibition of the detyrosination reaction was reported to decrease detyrosinated species [25]. This confirms one prediction of the CDT_N_ model for which the addition of an inhibitor of the detyrosination reaction (*k*_1_) can decrease the detyrosinated species and increase the tyrosination status (Fig 5D). These results are explained by the fact that the tyrosinated microtubule is a direct reactant of the detyrosination reaction (Fig 1A). The level of tyrosinated microtubule can therefore be increased by decreasing the reaction activity of its transformation into detyrosinated microtubule in proliferative and neuronal cells.

Our mathematical model CDT_N_ confirms that the inhibition of the TCP enzyme increases the tyrosination status in neuronal cells. Furthermore, the model predicts that the activation of two particular kinetic parameters, the tyrosination and detyrosinated microtubule depolymerization rate constants, in synergy, should suffice to enable an increase of the tyrosination status in living cells.

## Discussion

To our knowledge, the simple mathematical model presented here is the first mechanistic mathematical model of the tyrosination cycle and microtubules dynamics, developed in tight accordance with HCI experiments. The first expected outcome of such a mathematical model is to better understand the regulation mechanisms governing the microtubule tyrosination cycle, without considering all known and unknown microtubule-regulating proteins and their respective cross-talking signaling pathways.

Our generic model is parameterized for neurons and proliferating cells for which the observed microtubule behaviors and tyrosination status are different. Interestingly, our use of BIOCHAM optimization procedures with semi-qualitative semi-quantitative temporal logic constraints formalizing the experimental observations, showed that the minimal change in the parametrization of both models concerned two specific reaction kinetic parameter values only.

Our mathematical models provide fundamental mechanistic insights to better understand the microtubule dynamics and its dependence to the tyrosination status. They explain the incapacity to increase the tyrosination status by activating the tyrosination reaction alone due to the chain reactions present in the cycle and thus rationalize the failures of previous internal cell-based compound screening experiments. Moreover, the mathematical models predicted the inhibition of the detyrosination rather than the activation of the tyrosination has an effect on the tyrosination status in living cells and this prediction is experimentally validated in proliferative cells. Increasing the tyrosination status of microtubules may be achieved by a direct decrease of the TCP activity or its regulators because the association of TCP with microtubules is phosphorylation dependent [65,66].

Furthermore, several numerical analyses under perturbed conditions suggest a prospective drug combination as a novel approach to increase the tyrosination status of microtubules. The strategy is to increase the activity of the tyrosination and detyrosinated microtubule depolymerization reactions in synergy. TTL might be directly activated, although no drug was approved yet, or indirectly, via the inhibition of its inhibitors, since TTL activity is decreased through phosphorylation [35]. Another approach is to activate or inhibit, in synergy, signaling pathways that are known to modulate depolymerizing factors and involved in the modulation of the tyrosination cycle such as PKC, BDNF/TrkB, JNK, Stathmin pathways [66–70]. It is worth noting that these pathways are known to be dysregulated in neurodegenerative diseases [71–74].

Overall, our mechanistic-centric approach modeling the tyrosination cycle enhances the early drug research by providing critical mechanistic insights and identifying promising targets. We anticipate that our mathematical model should impact further investigations of the post-translational modifications of microtubules and their dysregulation in cancer, cardiomyopathies and other diseases [20–22]. The mathematical model aims at being extended beyond the cellular models presented in this paper. In particular, the inclusion of new parametrizations corresponding to other cell types for which dysregulation of the tyrosination cycle is associated with pathological conditions will be useful to expand its applications in drug discovery screening and cellular assay validation.

## Materials and methods

### Cell line models

We worked with hTERT RPE-1 cell line, an epithelial cell immortalized with hTERT from human retina (ATCC) and MEF cells, Mouse Embryonic Fibroblasts, produced by A. Andrieux’s group as proliferating cells. and CNS.4U neural cells (Ncardia) as hiPSC derived neurons. As hiPSC derived neurons cells we used CNS.4U neural cells (Ncardia).

### Culture method

hTERT RPE-1 cells were grown in DMEM/F12(1,1) supplemented with 10% fetal bovine serum and hygromycin (Life Technologies) 0.01 mg/ml in an atmosphere of 5% CO2 and 95% air at 37°C. Cells were maintained under standard conditions. For all experiments cells were plated overnight at 4 000 cells per well and 1 000 cells per well in CellCarrier-384 Ultra microplates (Perkin Elmer).

MEFs were thawed one week before experiment and grown in DMEM 1g/L Glucose supplemented with 1% Glutamax and 10% fetal bovine serum in an atmosphere of 5% CO2 and 95% air at 37°C. For all experiments cells were plated overnight at 2 500 cells per well and 2 000 cells per well in CellCarrier-384 Ultra microplates (Perkin Elmer).

CNS.4U cells were thawed directly in CellCarrier-384 Ultra microplates (Perkin Elmer) coated with poly-L-ornitin 0.01% (P4957, Sigma Aldrich) and laminin (1mg/mL), 7 days before experiment and they are maintained following suppliers’ instructions. In the first experiment with CNS.4U, cells were plated at a density of 6 000 cells/well and 9 000 cells/well in the second one.

### Cell free assay for TTL activators

A short biotinylated peptide corresponding to last 15 amino acids of the C-ter of tubulin was used as a substrat for TTL enzyme: Biotinyl-V-D-S-V-E-G-E-G-E-E-E-D-E-E. The enzyme TTL is pre-incubated with compound during 15min at room temperature. Then, the peptide substrat is added. Peptide was incubated at room temperature with different concentrations (10–100 μM) for TTL at a final concentration of 55nM in a reaction volume of 50μl (MES 50mM; KCl 100mM; MgCl2 25mM; DTT 0.001M; ATP 0.0003M et L-Tyrosine 0.001M in 5% DMSO) during 30min. After 30min, reaction was stopped by the addition of TFA to a final concentration of 1%. The final volume was about 100μl.

### Reaction products were analyzed by RapidFire/MS using the conditions described above

The RapidFire 365 (Agilent Technology) high-throughput system (RF) was coupled to a G-6460 triple quadrupole mass spectrometer (Agilent) operated in electrospray negative-ion mode. A type C cartridge was used for sample trapping and elution. The RapidFire method employs a solid phase extraction (SPE) sample cleanup step directly coupled to MS detection.

Samples were aspirated for 600 ms, followed by 4000 ms loading and washing with mobile phase A of 98% ddH2O+2%ACN+TFA0.01% at a flow rate of 1.5 mL/min. A fixed loop of 40 μL samples was loaded onto the cartridge. Samples were then eluted for 5000 ms with mobile phase B of 80% CAN + 20% ddH2O + TFA 0.01% at a flow rate of 1.25 mL/min, followed by reequilibrating the cartridge with mobile phase A at 0.7 mL/min for 500 ms.

MS parameters: Gas Temp: 200; Drying Gas: 9; Nebulizer: 40; Sheath Gas Temp: 400; Seath Gas Flow: 12; VCap: 3500; Nozzle Voltage: 300; Delta EMV: 400

MRM transitions for tubulin peptide substrat and reaction products were m/z 858.3 → 669.5, m/z 939.8 → 719.8, respectively. The dwell time for each transition was 4 ms. Peak areas were integrated, and the areas under curves are converted into the amount of substrate remaining and the product formed using a substrate and reaction product calibration curve.

### TCP inhibition with parthenolide combined with paclitaxel

MEF cells were plated overnight at 2 500 cells per well and 2 000 cells per well in CellCarrier-384 Ultra microplates (Perkin Elmer). Cells were distributed under 30 μl of medium. Next day plates were centrifuged before and after compounds adding at 900 rpm, 5 minutes, 20°C. Parthenolide was added with an ECHO 550 (Labcyte) in dose response from 1.7 to 50 μM in duplicate. After parthenolide, paclitaxel was added too in half microplates with ECHO 550 (Labcyte) at a final concentration of 5 μM. Compounds were incubated on cells 1h, 4h and 24h, then plates were fixed and labeled.

### Immunostaining

After incubation with compounds, cells were washed once with methanol and fixed 6 minutes with methanol (Fisher Medical) at room temperature. Cells were washed twice with PBS +/+ and permeabilized 15 minutes in PBS/BSA 2%/Triton 0,1%. After permeabilization solution aspiration, cells were incubated overnight at 4°C with a mixture of specific tubulin antibodies in PBS/BSA 2%/Triton 0,1%: YL1/2 (Tyr-tubulin antibody, Origene) and detyrosinated alpha Tubulin antibody (Abcam) at a 1/500 dilution, in some experiments a third primary antibody was used an anti-alpha tubulin (Abcam) at a 1/1000 dilution. Cells were rinsed twice in PBS +/+ and incubated 1 hour at room temperature with a mixture of secondary antibodies (Life Technologies) at a 1/1000 dilution in PBS/BSA 2%/Hoechst 33342 0,1%: Alexa Fluor 488 anti-mouse or anti-rat, Alexa Fluor 568 anti-rat or anti-rabbit and Alexa Fluor 647 anti-rabbit. Cells were washed twice in PBS +/+ and stayed in it.

Immunostaining was performed using the following primary antibody: anti-tyrosinated tubulin antibody (Abcam, Ab6160 1:1000) and anti-detyrosinated tubulin antibody (Abcam Ab48389 1:200) in a 0.5% triton solution. A single primary antibody has been used both for tyrosinated and detyrosinated tubulin ensuring the specificity and consistency of the analysis. The following secondary antibody were used: anti-rabbit Alexa fluor 488 antibody (Life A11034 1:1000), anti-rat Alexa fluor 647 (Life A21247 1:1000) and Hoechst (Life technologies H3570 1:1000) to stain for nuclei. We had to change the secondary antibody to adapt the different wavelengths for the imaging requirements depending on the cellular models. The choice of a secondary antibody does not change the primary antibody specificity and we did not compare the absolute intensity results between the experiments.

### High-content imaging and image analysis

High-content imaging descriptors measure the fluorescence level of different markers carried by antibodies that bind to biological species. Here, we are interested in tyrosinated (Tyr) and detyrosinated (Detyr) species in different cellular models. HCI descriptor values are measured and extracted at the single cell level through an image analysis (described below), which provides raw data for analysis. The major types of descriptors are shape-based, intensity-based, texture-based, and microenvironment-based [75]. In the current work, we are interested in intensity-based descriptors that are computed on actual intensity values in each channel of the image on a single-cell basis and HCI descriptor values corresponds to the intensity values of the markers averaged over a cell population.

Image acquisition was performed using either the Cell Voyager 8000 (Yokogawa Inc.) or the Opera Phenix (PerkinElmer, Waltham, MA, USA) high content screening systems. Images were captured using a 40X or 20X water immersion objective depending of the cell line and the cell morphologies.

Confocal images acquired by the Opera Phenix were then analyzed using the Harmony software (PerkinElmer) and those acquired by the Cell Voyager 8000 were first transferred to the Columbus Image Data Storage and analyzed using the Columbus Analysis System (PerkinElmer).

Since we used proliferative cells and neurons as cellular models, we had to optimize the image segmentation parameters to perform a consistent detection of the cellular compartments (nucleus and the cytoplasm) in these cellular models. To improve the nuclear segmentation, the nuclear staining channel image is first filtered using a regular Gaussian convolution kernel. Then, the nuclei segmentation is performed using the dedicated building block. To prevent false positive cell segmentations, nuclei were rejected at this stage based on morphological and intensity criteria. Using this filtered population of nuclei, the cytoplasm segmentation is performed using a region growing algorithm on a microtubule channel depending on the cellular model. Cells in contact with the edges of the image are discarded to prevent quantification on cropped cells, and finally signal intensity quantifications are performed on this selected cell population objects and exported at a single cell level.

### Modeling software biochemical abstract machine (BIOCHAM)

The Biochemical Abstract Machine (BIOCHAM) is a modelling software for cell systems biology based on Chemical Reaction Networks (CRN), with some unique features for static analysis, and dynamic analyses using temporal logic constraints. BIOCHAM is compatible with the Systems Biology Markup Language (SBML). BIOCHAM is a free software protected by the GNU General Public License GPL version 2 (http://lifeware.inria.fr/biocham4/). The online version 4.5.17 of BIOCHAM was used in this study.

### Parameter search procedures

Our CRN model of the detyrosination/tyrosination cycle (CDT) is interpreted by parametric Ordinary Differential Equations (ODE). The models CDT_N_ and CDT_P_ differ by the values of two reaction kinetic parameters.

The model CDT_N_ is parameterized with kinetic constants taken from the literature with some hypotheses or inferred from a parameter search procedure. The kinetic parameters *mc*_1_ and *mc*_2_ correspond to Michaelis constants for the detyrosinated and tyrosinated microtubule depolymerization reactions, and without direct experimental data, their values are inferred using BIOCHAM’s optimization procedure that aims at finding the values of (*mc*_1_, *mc*_2_) that make the tyrosinated species concentrations equal to 2.5 around five minutes knowing that their half-lives is of the order of minutes [58–60]. That constraint can be expressed in BIOCHAM by the following logical formula of First-Order Linear Time Logic with linear constraints over the reals, FO-LTL(Rlin), logic [52]:

F(Time==5/\Tyr=factor1)

and objective value *factor1 = 2*.*5*. The formula states that finally (***F***), at time around five units, the tyrosinated species concentration has some value assigned to variable *factor1*. Such a FO-LTL(Rlin) formula given with some objective values for its free variable (here *factor1*) can be evaluated on a simulation trace to give a continuous satisfaction degree in the interval [0,1] which indicates how far from satisfaction is the formula, between false (0) and true (1). Such a degree of satisfaction of the formal specification of the cell behaviour is used as objective function to guide search during parameter optimization.

BIOCHAM software uses the Covariance Matrix Adaptation Evolution Strategy (CMA-ES), a state-of-the-art black-box continuous non-linear optimization algorithm [76], to infer parameter sets satisfying such FO-LTL(Rlin) constraints [51,52].

In our study, we optimized the kinetic parameters *mc*_1_ and *mc*_2_ to parameterize the CDT_N_ model using the following BIOCHAM command:

    search_parameters(

        F(Time == 5 /\ Tyr = factor1),

        [0 <= mc1 <= 10, 0 <= mc2 <= 10],

        [factor1 -> 2.5]

    ).

The two different kinetic parameter values of the CDT_P_ model have been obtained by BIOCHAM’s optimization procedures for fitting the CDT_N_ model to experimental properties observed in proliferative cells and formalized in quantitative temporal logic [51,52]. The parameter search procedure aims at finding the minimal changes in the CDT_N_ model to make the system stabilize before five minutes and with a tenfold ratio of tyrosinated over detyrosinated species in accordance to our experimental and literature data. That constraint can be expressed in BIOCHAM by the following logical formula:

F(Time==5/\Tyr=ratio1*Detyr/\F(Time==20/\Tyr=ratio2*Detyr))

and objective values *ratio1 = ratio2 = 10*. The formula states that finally (***F***), at time around five units, the ratio Tyr over Detyr has some value assigned to variable *ratio1*, and later on (***F***) at time around 20, the ratio Tyr over Detyr has value *ratio2*. Such a FO-LTL(Rlin) formula given with some objective values for its free variables (here *ratio1* and *ratio2*) can be evaluated on a simulation (or experimental) trace to give a continuous satisfaction degree in the interval [0,1]. In our study, we optimized parameter values using the following BIOCHAM command schema, given below for two parameters (*V*_*m*2_, *k*_1_):

    search_parameters(

        F(Time == 5 /\ Tyr = factor1 * Detyr

            /\ F(Time == 20 /\ Tyr = factor2 * Detyr)),

        [0<= Vm2 <= 1000, 0 <= k1 <= 100],

        [factor1 -> 10, factor2 -> 10]

    ).

### Robustness measure procedure

In BIOCHAM, the continuous satisfaction degree of an FO-LTL(Rlin) formula, evaluated on a given simulation (or experimental) trace, and given with objective values for the free variables, is used to guide the search to compute (locally) optimal parameter values, but also to compute parameter sensitivity indices and model robustness measures with respect to parameter perturbation [52]. In BIOCHAM, parameter sensitivity indices are computed by estimating the mean satisfaction degree (i.e. robustness [77]) of the temporal specification by varying each parameter independently [52]. We computed the sensitivity indices of the ratio between tyrosinated and detyrosinated species (TyrDetyr) at steady state (given here by value 0.065386 for that ratio) to variations of the reaction kinetic parameters by a coefficient of 100%, such as the detyrosination rate constant (*k*_1_) below, using the following BIOCHAM command:

    sensitivity(

        F(G(TyrDetyr = x)),

        [k1],

        [x -> 0.065386],

        robustness_coeff_var: 1

    ).

### Landscape of behavioural constraint satisfaction degree

The following BIOCHAM command is used to obtain the landscape of Fig 2E:

    scan_parameters(

        F(Time == 5 /\ Tyr = factor1 * Detyr /\ F(Time == 20 /\ Tyr == factor2 * Detyr)),

        (0 <= Vm2 <= 15),

        (0 <= km1 <= 30),

        [factor1 -> 10, factor2 -> 10],

        resolution:30).

This command returns some statistics and draws the landscape of the satisfaction degrees (truncated to 1) of a FO-LTL(Rlin) formula obtained by varying two parameters in given intervals. Here, the FO-LTL(Rlin) formula is: F(Time == 5 /\ Tyr = factor1 * Detyr /\ F(Time == 20 /\ Tyr = factor2 * Detyr)) and the kinetic parameters (*V*_*m*2_, *k*_*m*1_) are scan within the specified intervals (0–15, 0–30) respectively.

### Multistability analysis

BIOCHAM software can check necessary conditions for the existence of multiple non-degenerate steady states in the differential dynamics of a reaction model, by checking the existence of positive circuits in a labeled influence graph associated to the structure of the reaction network [78].

When applied to our reaction model, the BIOCHAM command:

    check_multistability.

returns the absence of such positive circuits. This proves the absence of multiple non degenerate steady states in the ODE system and ensure the uniqueness of the stable state reached from different initial conditions.

### Unperturbed numerical simulation

Unperturbed numerical simulations correspond to the numerical simulations of the computational models CDT_N_ and CDT_P_ as parameterized. Numerical simulations of the computational models CDT_N_ and CDT_P_ were carried out within BIOCHAM.

BIOCHAM reaction models can be interpreted in multiple ways. In the differential semantics, we can run the software command *numerical_simulation*. That command performs a numerical integration from time 0 up to a given time *x* specified with the command *option(time*:*x)*.

By default, BIOCHAM uses the numerical solver *bsimp (Implicit Bulirsch-Stoer method of Bader and Deuflhard) of* the GNU Scientific Library to perform numerical simulations. BIOCHAM offers other numerical solvers for continuous, stochastic and boolean models not used in this study.

Numerical simulations results were exported in csv format file using the command *export_plot(“FILENAME”)*. The resulting csv file was downloaded and copied pasted within the software GraphPad 8.3.0 for a better rendering of figures. The landscape figures were directly produced by BIOCHAM.

### Perturbed numerical simulation

We simulated the effect of adding an activator or an inhibitor compound in the parameterized computational models CDT_N_ or CDT_P_ by increasing or decreasing kinetic parameters for a given time condition.

In BIOCHAM, perturbed numerical can be achieved by using events to change some parameter values during simulation once a condition becomes satisfied. A condition can be based on time (variable *Time*) or on molecular species values. For instance, when time becomes greater than 20 (units of time) in a numerical simulation, we decreased or increased the value of some kinetic parameters, hence the name perturbed numerical simulation.

We performed several perturbed numerical simulations using the computational models CDT_N_ and CDT_P_. The BIOCHAM command:

        add_event(Time > 20, k1 = 0.01)

specifies that when the time becomes greater than 20 (unit of time), the kinetic parameter *k*_1_, i.e. the detyrosination rate constant, is set to 0.01. This command simulates the addition of an inhibitor of the detyrosination reaction at time 20.

We also performed several perturbed numerical simulations using the unperturbed computational models CDT_N_ and CDT_P_ to simulate prospective drug combinations. The commands

        add_event(Time > 20, km1 = 4.78, Vm2 = 2)

    and

      add_event(Time > 20, km1 = 0.478, Vm2 = 0.2)

specify changes of values of the depolymerization rate constant of detyrosinated microtubule, *k*_*m*1_, and the tyrosination rate constant, *V*_*m*2_, at next time greater than 20 (time units), and then later on at next time greater that 20.

In our study, we performed the following perturbed numerical simulations for simulating:

- the addition of an activator of TTL, by increasing the tyrosination rate constant (*V*_*m*2_),
- the addition of an inhibitor of TCP, by decreasing the detyrosination rate constant (*k*_1_),
- the addition of an activator of the depolymerization factors, by increasing the detyrosinated microtubule depolymerization rate constant (*k*_*m*1_),
- the addition of an activator of the depolymerization factors synergistically combined with the addition of an activator of TTL, by increasing both the detyrosinated microtubule depolymerization rate constant (*k*_*m*1_) and the tyrosination rate constant (*V*_*m*2_).

### Data analysis

The data processed in the study come from numerical simulations, parameter search and sensitivity analysis using BIOCHAM software (unperturbed and perturbed numerical simulations), raw data from the image analysis which were processed by software Colombus and Harmony.

The data from numerical simulations, sensitivity analysis and parameter scan were uploaded and processed within GraphPad 8.3.0 software for the creation of figures.

For all datasets exported from image analysis from Columbus and Harmony, python scripts have been developed to extract the HCI descriptors of interest in order to export the data in csv format file to be used by GraphPad 8.3.0 software. The HCI descriptors of interest are those representing the quantification of Tyr, Detyr and the Tyr/Detyr ratio in the respective experiments. The Tyr/Detyr ratio has been computed during the image analysis process or directly from the HCI descriptors Tyr and Detyr within the scripts.

From processed data from image analysis, GraphPad 8.3.0 software was used for the creation of figures, to compute descriptive statistics and for curve fitting. The Z’- factor displayed within GraphPad figure was computed within Excel sheet using the Z’-factor formula described above.

### Statistical analysis

In high-content imaging, the Z’-factor criteria is classically used in order to evaluate the quality of chosen readout and judge the response quality of a particular assay [79]. The Z’-factor indicates the extent of separation between positive and negative. It is defined as:

$$Z’-factor=1-\frac{3(\sigma_{p}+\sigma_{n})}{\left|\mu_{p}-\mu_{n}\right|}$$

where *μ*_*p*_ and *σ*_*p*_ are the mean and standard deviation of the positive control (or alternately, the treated samples) and *μ*_*n*_ and *σ*_*n*_ are those of the negative control.

The Z’-factor ranges from -∞ to 1, with the following interpretation:

- 1 > Z’ > = 0.5: statistical distribution from the negative and positive controls are well separated: separation band is large
- 0.5 > Z’ > = 0: statistical distribution from the negative and positive controls are separated: separation band is small
- Z’ < 0: statistical distribution from the negative and positive controls overlap: no separation band, the negative control signal variation and the positive control signal variation bands overlap

## Supporting information

S1 Fig Plate layout for tyrosination status quantification and detyrosination reaction inhibition in MEF cells and hTERT cells. MEF and hTERT RPE-1 cells were screening using 5 compounds in dose response and kinetic. Compound names: Parthenolide, IB737, IBZ36, IBZ35, Paclitaxel. Negative control: DMSO. Positive control: Paclitaxel at 5 μM. Dose response range for the screened compounds: from 1.7 μM to 50 μM in duplicate. Incubation time: 1 hour, 4 hours and 24 hours. Antibodies: Tub Tyr (Origen, SM2202P) + A488, Tub deTyr (Abcam, ab48389) + A647. Following treatment, an image analyses was performed (see Materials and Methods). Following image analysis, raw data were processed (see Materials and Methods). The HCI descriptors Tyr, Detyr, Tyr/Detyr were used for data analysis. The tyrosination status quantification data analysis used the wells from the negative controls at 1 hour. The detyrosination reaction inhibition data analysis used the wells were Parthenolide and Parthenolide+Paclitaxel were screened in dose response at 1 hour and the wells from the negative and positive controls.
(TIF)

S2 Fig Example of compound activities on the tyrosination status of tubulin C-terminals in cell free assay. Dose response diagrams in replicates (n1, n2) showing an increase of the tyrosination status (% Effect) of tubulin C-terminals by activation of the TTL enzyme in cell free assay. Data in each graph were fit using a sigmoidal dose-response curve with the ExcelFit software. Compound EC50 values in μM: (Compound 1: 1.63E-05 (n1), >3.00E-05 (n2)), (Compound 2: 6.77E-06 (n1), 6.77E-06 (n2)), (Compound 3: 6.83E-06 (n1), 6.09E-06 (n2)), (Compound 4, 2.98E-06 (n1), 3.31E-06 (n2)). The X axis in each graph is presented as log10 values.
(TIF)

S3 Fig Plate layout for compound screening in MEF cells and CNS.4U cells. MEF and CNS.4U cells were screening using 4 chemical compounds in dose response and kinetic. Compound names: Compound 1, Compound 2, Compound 3, Compound 4. Negative control: DMSO. Positive control: Paclitaxel at 5 μM. Dose response range for the screened compounds: from 0.3 μM to 10 μM in duplicate. Incubation time: 5 min. Antibodies: Tub Tyr (Life Technologies, A11077) + A568, Tub deTyr (Life Technologies, A21245) + A647. Following treatment, an image analyses was performed (see Materials and Methods). Following image analysis, raw data were processed (see Materials and Methods). The HCI descriptor Tyr/Detyr were used for data analysis. The data analysis for compound screening in MEF and CNS.4U cells used all the wells.
(TIF)

S4 Fig High-content imaging descriptor Tyr/Detyr from compound screening in MEF cells. MEF cells were screening using 4 compounds in dose response and kinetic. Compound names: Compound 1, Compound 2, Compound 3, Compound 4. Negative control: DMSO. Dose response range for the screened compounds: from 0.3 μM to 10 μM in duplicate. At any dose, the tyrosination status did not significantly increased (Z’ < 0.5). Incubation time: 1 hour. Following treatment, an image analyses was performed (see Materials and Methods). Following image analysis, raw data were processed (see Materials and Methods). The HCI descriptor Tyr/Detyr was extracted and fit using a sigmoidal dose-response curve to the data, using the GraphPad Prism 8.3.0 software. Log IC50 values in μM: (Compound 1, -3.354), (Compound 2, Not converged), (Compound 3, -5.121), (Compound 4, -5.391). DMSO values were interpolated using a line curve. The X axis in each graph is presented as log10 values, and the data are plotted as the mean ± SD.
(TIF)

S5 Fig High-content imaging descriptor Tyr/Detyr from compound screening in CNS.4U cells. CNS.4U cells were screening using 4 compounds in dose response and kinetic. Compound names: Compound 1, Compound 2, Compound 3, Compound 4. Negative control: DMSO. Dose response range for the screened compounds: from 0.3 μM to 10 μM in duplicate. At any dose, the tyrosination status did not significantly increased (Z’ < 0.5). Incubation time: 5 minutes. Following treatment, an image analyses was performed (see Materials and Methods). Following image analysis, raw data were processed (see Materials and Methods). The HCI descriptor Tyr/Detyr was extracted and fit using a sigmoidal dose-response curve to the data, using the GraphPad Prism 8.3.0 software. Log IC50 values in μM: (Compound 1, -6.818), (Compound 2, -5.905), (Compound 3, -5.732), (Compound 4, -6.29). DMSO values were interpolated using a line curve. The X axis in each graph is presented as log10 values, and the data are plotted as the mean ± SD.
(TIF)

S1 Data High-content imaging data for tyrosination status quantification and detyrosination reaction inhibition in hTERT RPE-1 cells and MEF cells. (ZIP)

S2 Data High-content imaging data from compounds screening in MEF cells and CNS.4U cells. (ZIP)
