# Supplementary figures and images for: Mathematical modeling of the microtubule detyrosination/tyrosination cycle for cell-based drug screening design
Source: PLoS Comput Biol. 2022 Jun 27;18(6):e1010236. doi: 10.1371/journal.pcbi.1010236 (PMC9236252; doi:10.1371/journal.pcbi.1010236)

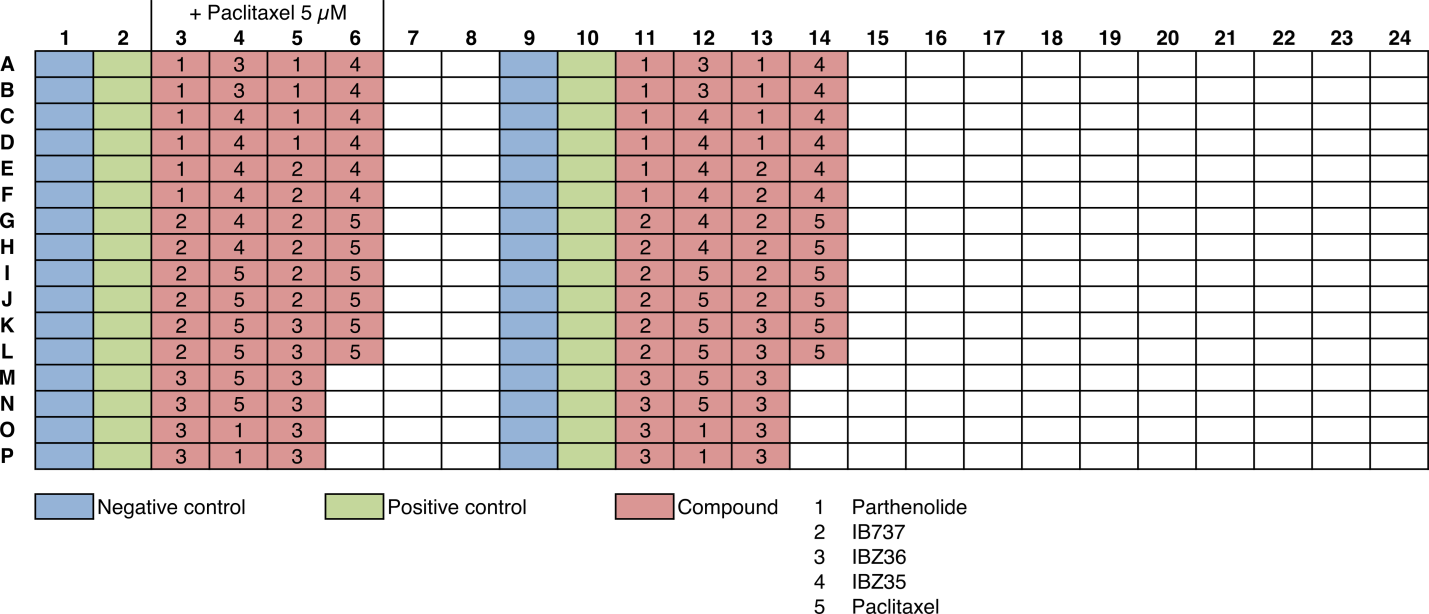

Supplement: S1 Fig — MEF and hTERT RPE-1 cells were screening using 5 compounds in dose response and kinetic. Compound names: Parthenolide, IB737, IBZ36, IBZ35, Paclitaxel. Negative control: DMSO. Positive control: Paclitaxel at 5 μM. Dose response range for the screened compounds: from 1.7 μM to 50 μM in duplicate. Incubation time: 1 hour, 4 hours and 24 hours. Antibodies: Tub Tyr (Origen, SM2202P) + A488, Tub deTyr (Abcam, ab48389) + A647. Following treatment, an image analyses was performed (see Materials and Methods). Following image analysis, raw data were processed (see Materials and Methods). The HCI descriptors Tyr, Detyr, Tyr/Detyr were used for data analysis. The tyrosination status quantification data analysis used the wells from the negative controls at 1 hour. The detyrosination reaction inhibition data analysis used the wells were Parthenolide and Parthenolide+Paclitaxel were screened in dose response at 1 hour and the wells from the negative and positive controls. (TIF) [file pcbi.1010236.s001.tif]

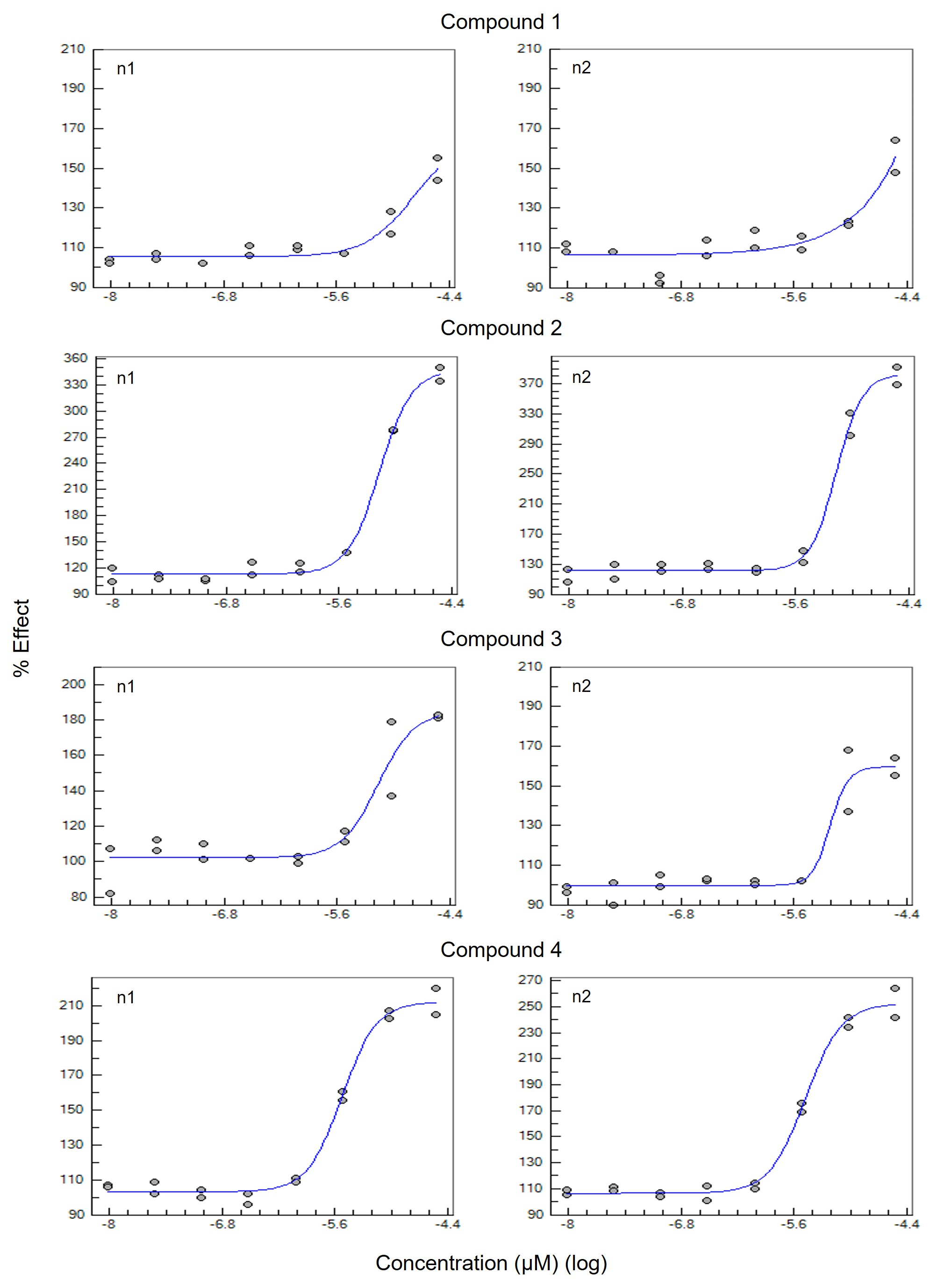

Supplement: S2 Fig — Dose response diagrams in replicates (n1, n2) showing an increase of the tyrosination status (% Effect) of tubulin C-terminals by activation of the TTL enzyme in cell free assay. Data in each graph were fit using a sigmoidal dose-response curve with the ExcelFit software. Compound EC50 values in μM: (Compound 1: 1.63E-05 (n1), >3.00E-05 (n2)), (Compound 2: 6.77E-06 (n1), 6.77E-06 (n2)), (Compound 3: 6.83E-06 (n1), 6.09E-06 (n2)), (Compound 4, 2.98E-06 (n1), 3.31E-06 (n2)). The X axis in each graph is presented as log10 values. (TIF) [file pcbi.1010236.s002.tif]

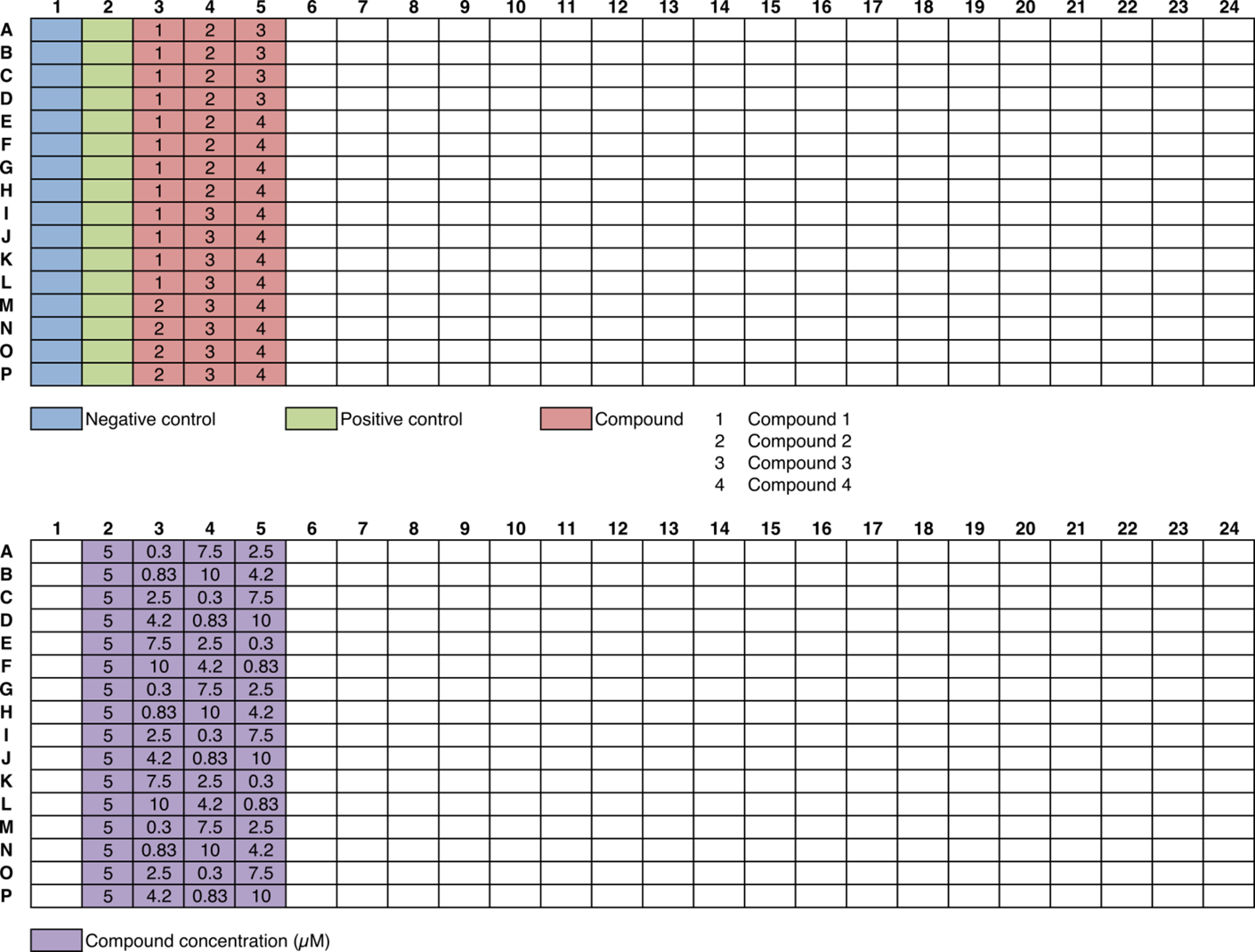

Supplement: S3 Fig — MEF and CNS.4U cells were screening using 4 chemical compounds in dose response and kinetic. Compound names: Compound 1, Compound 2, Compound 3, Compound 4. Negative control: DMSO. Positive control: Paclitaxel at 5 μM. Dose response range for the screened compounds: from 0.3 μM to 10 μM in duplicate. Incubation time: 5 min. Antibodies: Tub Tyr (Life Technologies, A11077) + A568, Tub deTyr (Life Technologies, A21245) + A647. Following treatment, an image analyses was performed (see Materials and Methods). Following image analysis, raw data were processed (see Materials and Methods). The HCI descriptor Tyr/Detyr were used for data analysis. The data analysis for compound screening in MEF and CNS.4U cells used all the wells. (TIF) [file pcbi.1010236.s003.tif]

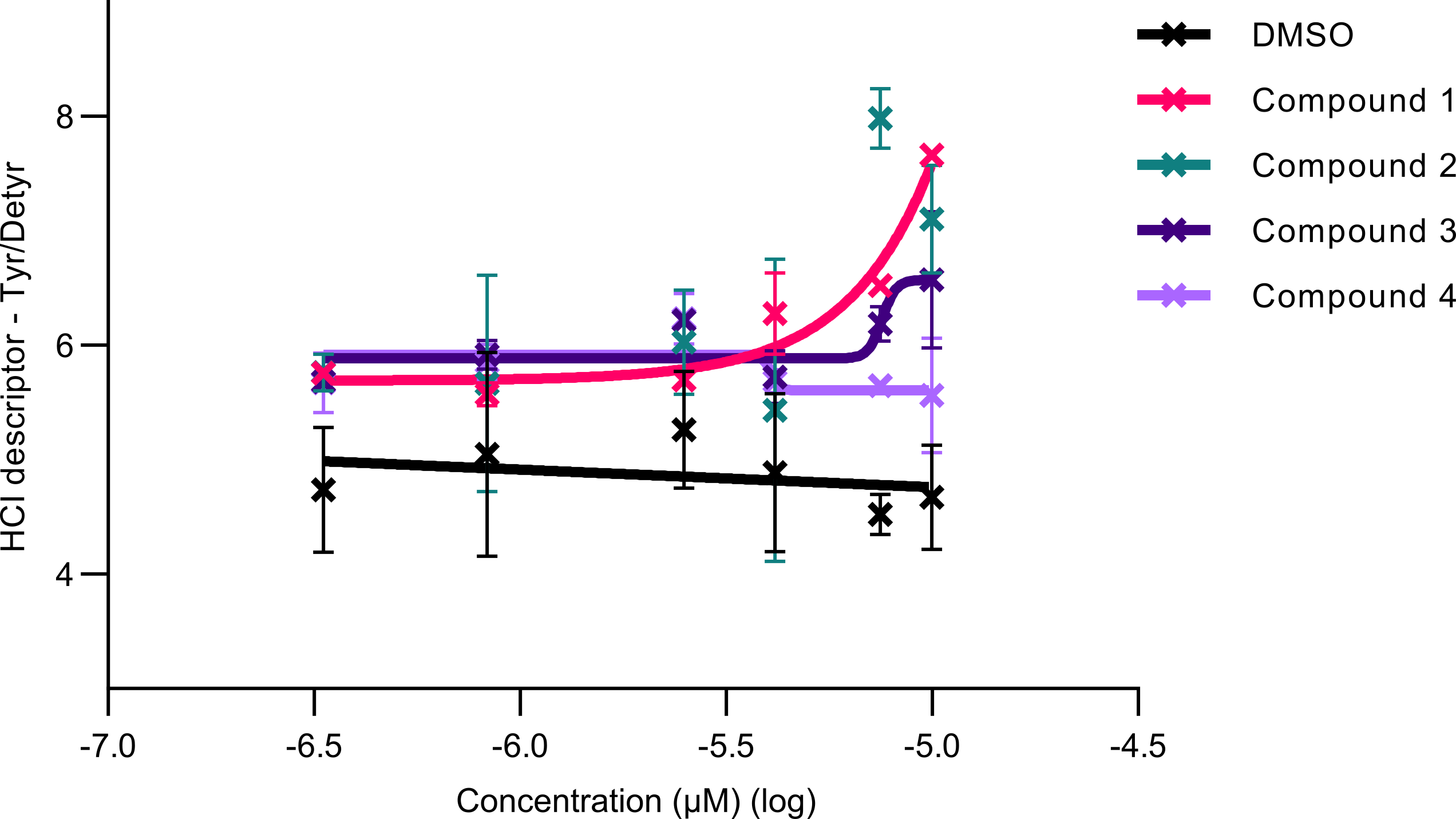

Supplement: S4 Fig — MEF cells were screening using 4 compounds in dose response and kinetic. Compound names: Compound 1, Compound 2, Compound 3, Compound 4. Negative control: DMSO. Dose response range for the screened compounds: from 0.3 μM to 10 μM in duplicate. At any dose, the tyrosination status did not significantly increased (Z’ < 0.5). Incubation time: 1 hour. Following treatment, an image analyses was performed (see Materials and Methods). Following image analysis, raw data were processed (see Materials and Methods). The HCI descriptor Tyr/Detyr was extracted and fit using a sigmoidal dose-response curve to the data, using the GraphPad Prism 8.3.0 software. Log IC50 values in μM: (Compound 1, -3.354), (Compound 2, Not converged), (Compound 3, -5.121), (Compound 4, -5.391). DMSO values were interpolated using a line curve. The X axis in each graph is presented as log10 values, and the data are plotted as the mean ± SD. (TIF) [file pcbi.1010236.s004.tif]

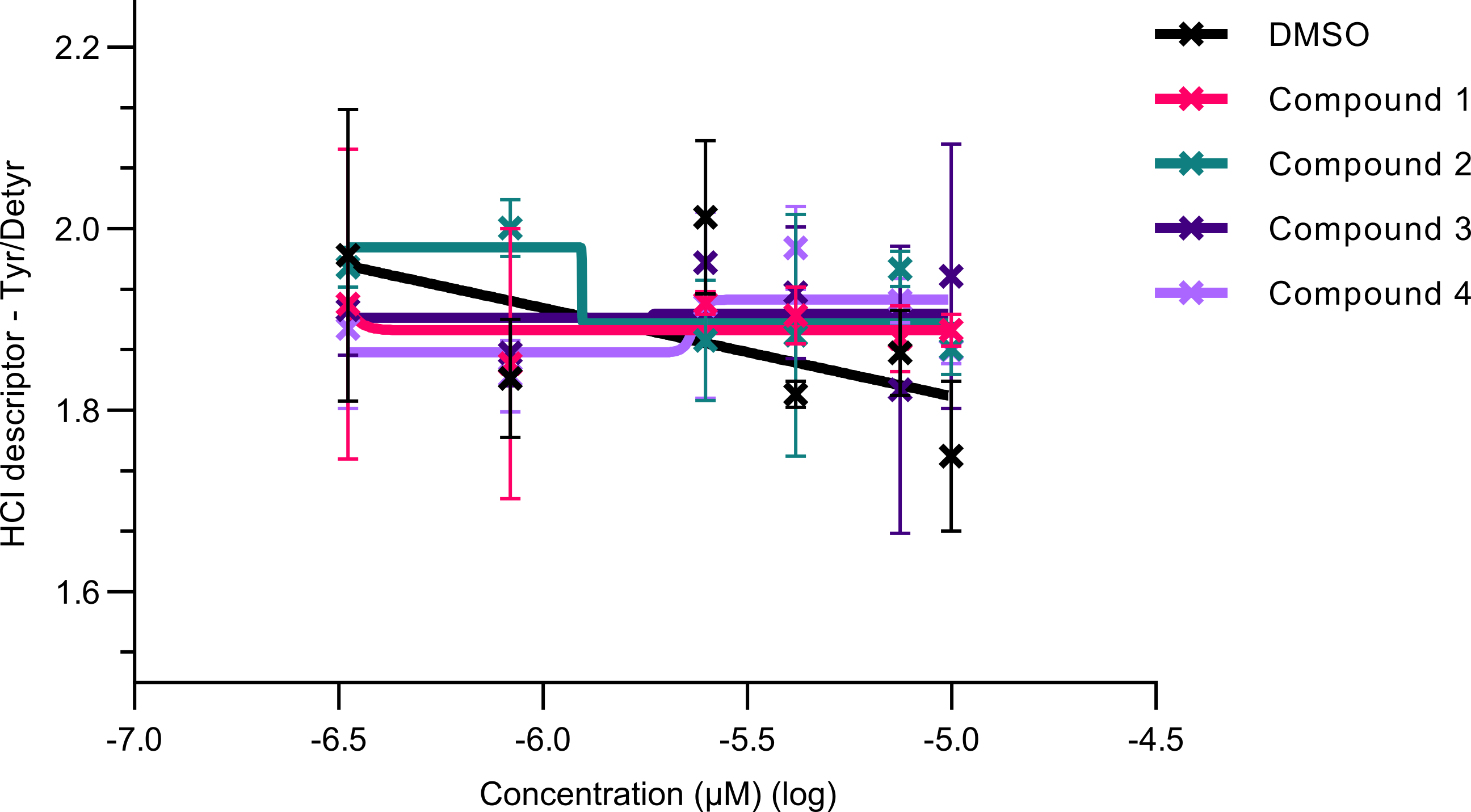

Supplement: S5 Fig — CNS.4U cells were screening using 4 compounds in dose response and kinetic. Compound names: Compound 1, Compound 2, Compound 3, Compound 4. Negative control: DMSO. Dose response range for the screened compounds: from 0.3 μM to 10 μM in duplicate. At any dose, the tyrosination status did not significantly increased (Z’ < 0.5). Incubation time: 5 minutes. Following treatment, an image analyses was performed (see Materials and Methods). Following image analysis, raw data were processed (see Materials and Methods). The HCI descriptor Tyr/Detyr was extracted and fit using a sigmoidal dose-response curve to the data, using the GraphPad Prism 8.3.0 software. Log IC50 values in μM: (Compound 1, -6.818), (Compound 2, -5.905), (Compound 3, -5.732), (Compound 4, -6.29). DMSO values were interpolated using a line curve. The X axis in each graph is presented as log10 values, and the data are plotted as the mean ± SD. (TIF) [file pcbi.1010236.s005.tif]
